# Selective Adsorption and Purification of the Acteoside in *Cistanche tubulosa* by Molecularly Imprinted Polymers

**DOI:** 10.3389/fchem.2019.00903

**Published:** 2020-01-23

**Authors:** Xiaobin Zhao, Wenjing Pei, Ruili Guo, Xueqin Li

**Affiliations:** Key Laboratory for Green Processing of Chemical Engineering of Xinjiang Bingtuan, School of Chemistry and Chemical Engineering, Shihezi University, Shihezi, China

**Keywords:** molecularly imprinted polymer, *Cistanche tubulosa*, acteoside, adsorption, hydrogen bond

## Abstract

Acteoside (ACT) is the main component of phenylethanoid glycosides in *Cistanche tubulosa*, and it is extremely desirable for obtaining high purification of ACT by molecularly imprinted polymers (MIPs) from their extracts. In this study, MIPs were designed and synthetized to adsorb selectively the ACT in *C. tubulosa*. The effects of different functional monomers, cross-linkers, and solvents of MIPs were investigated. MIPs were studied in terms of static adsorption experiments, dynamic adsorption experiments, and selectivity experiments. The optimal functional monomer, cross-linking agent, and solvent are 4-vinylpyridine, ethylene glycol dimethylacrylate, and the mixed solvent (acetonitrile and *N,N*-dimethylformamide, 1:1.5, v/v), respectively. Under the optimal conditions, the synthesized MIP1 has a high adsorption performance for ACT. The adsorption capacity of MIP1 to ACT reached 112.60 mg/g, and the separation factor of ACT/echinacoside was 4.68. Because the molecularly imprinted cavities of MIP1 resulted from template molecules of ACT, it enables MIP1 to recognize selectively ACT. Moreover, the N–H groups on MIP1 can form hydrogen bonds with the hydroxyl groups on the ACT; this improves the separation factor of MIP1. The dynamic adsorption of ACT accorded with the quasi-second-order kinetics; it indicated that the adsorption process of MIP1 is the process of chemical adsorption to ACT. MIPs can be applied as a potential adsorption material to purify the active ingredients of herbal medicines.

## Introduction

*Cistanche tubulosa* is one of the valuable Chinese herbal remedies (Li et al., 2016; Morikawa et al., 2019). The crude extract of *C. tubulosa* mainly contains phenylethanoid glycosides, polysaccharides, oligosaccharides, flavonoids, polyphenols, and proteins, among which phenylethanoid glycosides are the most effective components of *C. tubulosa* (Wang et al., 2015, 2017; Yan et al., 2017). Studies have shown that phenylethanoid glycoside compounds have the effects of tonifying kidney Yang, anti-oxidation, anti-tumor, anti-aging, enhancing memory, etc. Phenylethanoid glycosides have a wide range of applications in medicine, health care, food, and so on (Yang et al., 2017; Fu et al., 2018; Wu et al., 2019; Xu et al., 2019). In order to obtain high-purity products, the highly selective separation of the target substance is the key to the problem. Therefore, it is of great significance to design and develop a purification method with good selectivity, high efficiency, energy saving, and environmental friendliness for the development and utilization of *C. tubulosa*.

Phenylethanoid glycosides are the most important pharmacological active ingredients in *C. tubulosa*, including echinacoside (ECH), acteoside (ACT), isomeric, and 2-acetyl *C. tubulosa* glycosides. ECH and ACT are the main components of phenylethanoid glycosides, with content up to 90%. At present, the research of *C. tubulosa* mainly focuses on its pharmacological components. The separation and purification methods mainly include macroporous adsorption resin, high-speed countercurrent chromatography, membrane separation, and molecular imprinting (Han et al., 2012; Dong et al., 2015; Zhang et al., 2018a; Pei et al., 2019; Si et al., 2019). Macroporous adsorption resin is a mature technology, but there are some shortcomings such as a large amount of solvent, long time, low yield, and complex process. High-purity monomer compounds can be extracted from natural products by high-speed countercurrent chromatography. Membrane separation is a new kind of separation technology, which can make the effective components of natural products rich and with few impurities, but the separation process is complicated (Li et al., 2015a,b; Wang et al., 2016; Zhang et al., 2016, 2018b; Li X. et al., 2019). As a new separation technique, molecular imprinting can make the active components of natural products highly concentrated with few impurities and effectively improve the purity of products. Molecularly imprinted polymers (MIPs) have shown the important applications for the purification and preconcentration of biomolecules from complex human fluids such as urine or postmortem blood (Lulinski et al., 2015, 2016).

Molecular imprinting technology synthesizes highly cross-linked MIPs through template orientation, generating cavities that mimic binding sites of antibodies, enzymes, and other biological materials, and give priority to bind with template molecules, providing an effective method for molecular recognition (Hrobonova et al., 2018; Liang et al., 2018; Hong et al., 2019; Ma et al., 2019). MIPs have attracted wide attention in the fields of solid phase extraction, sensors, antibodies, enzyme simulation, receptors, and catalysts (Zhang et al., 2013; Ansari and Karimi, 2017; Diltemiz et al., 2017; Xiao et al., 2018; Yu et al., 2019). Recently, MIPs have potential applications in drug delivery devices or chiral resolution (Lulinski, 2017; Marc et al., 2018; BelBruno, 2019; Sobiech et al., 2019). The main advantages of MIPs are the ease of preparation and creation of “custom” possible binding sites, by adjusting the target molecule's synthesis process needed as a template in the polymerization process, as well as the advantages of low production cost, stability, robustness, and acid and alkali resistance (Speltini et al., 2017; Wu et al., 2017; Xu et al., 2017; Li F. et al., 2019; Zhang et al., 2019). In particular, MIPs have been successfully used as a selective adsorbent for solid phase extraction to extract active ingredients from natural products (Huang et al., 2019; Li Z. et al., 2019; Wang Y. et al., 2019). Molecular imprinting is divided into covalent molecular imprinting and non-covalent molecular imprinting. Covalent molecular imprinting has the characteristics of strong adhesion and difficult elution of template molecules, while non-covalent molecular imprinting has the characteristics of strong adhesion and easy elution of template molecules. Therefore, non-covalent molecular imprinting polymers are often used for the separation and purification of natural products. The binding mode of non-covalent molecular imprinting method and target components is generally weak covalent bond binding, such as hydrogen bond, van der Waals force, hydrophobic interaction, π-π accumulation (Yoshikawa et al., 2016; Vicario et al., 2018), etc. The recent literatures have reported that the interaction between the components of prepolymerization complex can be discussed by the theoretical analysis for design of functional monomers, cross-linking agents, and solvents (Sobiech et al., 2014, 2017; Cowen et al., 2016; Giebultowicz et al., 2019). Precipitation polymerization is the most commonly used method in the synthesis of imprint materials, but the main disadvantage of this method is that the steps required for the preparation of imprint materials are complex and numerous (Phungpanya et al., 2018). Therefore, this study mainly obtained a kind of imprinted material with high selective adsorption capacity for ACT by bulk polymerization, which is a simple and rapid synthesis method (Cantarella et al., 2019; Wang H. et al., 2019).

The aim of this study is to obtain an imprinted material with high selective adsorption capacity for ACT by a simple and rapid synthesis method. A series of MIPs of different functional monomers and different solvents were synthesized by bulk polymerization. The synthetic materials were characterized by scanning electron microscopy (SEM) and Fourier transform infrared spectroscopy (FT-IR). The adsorption performance of phenylethanoid glycoside aqueous solution was evaluated, and its binding selectivity was studied in depth. The MIPs with optimal adsorption performance were used to adsorb and purify ACT from crude extract of *C. tubulosa*.

## Materials and Methods

### Materials

Echinacoside (ECH, ≥98%) and Acteoside (ACT, ≥98%) were obtained from Sunny Biotech Co., Ltd. (Shanghai, China). *C. tubulosa* was obtained from Cistanche Rongtang Biotechnology Co., Ltd. (Xinjiang, China). 4-Vinylpyridine (4-VP, 98%), methacrylic acid (MAA, 98%), 2-hydroxyethyl methacrylate (HEMA, 98%), ethyleneglycol dimethacrylate (EGDMA, 98%), azodiisobutyronitrile (AIBN, 98%), divinylbenzeneare (DVB, 98%) and *N, N*-dimethylformamide (DMF, 99.5%) were obtained from Adamas Reagent Co., Ltd. (Shanghai, China). Acetonitrile (ACN, ≥99.9%), methanol (≥99.9%), and acetic acid (≥99.9%) were obtained from ThermoFisher Scientific Co., Ltd. (Shanghai, China). Ethanol (≥99.7%) was obtained from Yong sheng Fine Chemical Co., Ltd. (Tianjin, China). Deionized water is prepared from laboratory pure water Smart-S15 system (Shanghai, China).

### Instruments

The surface morphology and microstructure were examined by scanning electron microscopy (SEM, SU8010, Hitachi, Japan). The chemical structure of MIPs (FT-IR, icolet AVATAR360, Nikolai, USA) was measured by FT-IR. FT-IR test conditions: the step length is 2 cm^−1^ and the scanning range is 4,000–500 cm^−1^, and attenuated total reflection method is used to prepare the MIP. The pore size, distribution and specific surface area of MIPs are measured using a special physical adsorption device (Mike, ASAP 2460). Adsorbent test conditions: degassing at 60°C for 12 h, N_2_ adsorption and desorption curves were measured at −196°C. ^1^H nuclear magnetic resonance (^1^H NMR) spectra were recorded in DMSOd6 on an AV-300 spectrometer (Bruker, Switzerland) with TMS as internal standard and values are shown in ppm (δ).

High-performance liquid chromatography (HPLC) with ultraviolet (UV) ray detector was performed with a 2695 solution system (Waters, USA). A chromatography was performed on a reverse-phase C18 column (Symmetry, 250 × 4.6 mm, 5 μm). The analytical methods were as follows: the mobile phase was acetonitrile (A) and acetic acid/water (1:44, v/v) (B) at a flow rate of 1 ml/min with 10-μl injection volumes and the UV detector wavelength was set at 330 nm. The column temperature was maintained at 30°C (Yang et al., 2018). Gradient elution conditions are listed in Table 1.

**Table 1:** The gradient elution program of HPLC.

***T* (min)**	**A (%)**	**B (%)**
0–8	13	87
8–20	13–20	87–80
20–23	20–50	80–50
23–25	50–13	50–87
25–28	13	87

HPLC analysis using ECH and ACT standard solutions (2 mg/ml, 1 mg/ml, 0.2 mg/ml, 0.04 mg/ml, 0.008 mg/ml, and 0.0016 mg/ml) gave ECH and ACT calibration. The linear regression equation is shown in Table 2.

**Table 2:** Calibration curves of ECH and ACT.

**Component**	**Regression equation**	***R*^**2**^**	**Linear range (μg/ml)**
ECH	*Y* = 1.32 × 10^7^*X* – 1.50 × 10^4^	0.999988	1.6–2,000
ACT	*Y* = 1.71 × 10^7^*X* – 1.57 × 10^3^	0.999987	1.6–2,000

The limits of detection for ACT and ECH were 0.528 and 0.528 μg/ml, respectively. The limits of quantification for ACT and ECH were 1.60 and 1.60 μg/ml, respectively. The accuracy of ACT and ECH were 1.40 and 1.89%, respectively. The same sample was injected five times to obtain ACT and ECH precision of 1.40 and 1.71%, respectively.

### Synthesis of MIPs

The preparation process of MIP1 as follows: firstly, the template molecule ACT (125.00 mg) and the functional monomer 4-VP (210.00 mg) were dissolved sufficiently in mixed solution (2.50 ml) of acetonitrile and *N,N*-dimethylformamide (1:1.5, v/v). Then, the prepolymerization reaction of the mixture was carried out at 25°C for 20 min. Subsequently, EGDMA (1.12 g) and AIBN (15.00 mg) were added and dissolved fully into the pre-polymerization mixture. The obtained prepolymer solution was evacuated and filled with argon gas. The process of polymerization was carried out at 60°C for 24 h. Finally, the pale-yellow bulk polymers were obtained and grounded to powder, which was sieved through a 200-mesh screen.

The mixed solution of methanol and acetic acid (9:1, v/v) was used to elute template molecules of ACT. The ACT molecules were eluted and repeated to wash until no ACT molecules were found in MIP1. The residual acetic acid in MIP1 was washed with methanol, and then MIP1 were dried at 40°C.

ACT was used as the template molecule, and different functional monomers, cross-linkers, and solvents are listed in Table 3 and used to synthesize MIPs and NIPs.

**Table 3:** Ratio of raw materials for molecularly imprinted polymerization.

**Polymers**	**Template**	**Functional monomer**	**Cross-linker**	**Solvent**
MIP1	ACT	4-VP	EGDMA	ACN:DMF (1.5:1, v/v)
MIP2	ACT	MAA	EGDMA	ACN:DMF (1.5:1, v/v)
MIP3	ACT	HEMA	EGDMA	ACN:DMF (1.5:1, v/v)
MIP4	ACT	4-VP	DVB	ACN:DMF (1.5:1, v/v)
MIP5	ACT	4-VP	EGDMA	Methanol
NIP1	-	4-VP	EGDMA	ACN:DMF (1.5:1, v/v)
NIP2	-	MAA	EGDMA	ACN:DMF (1.5:1, v/v)
NIP3	-	HEMA	EGDMA	ACN:DMF (1.5:1, v/v)
NIP4	-	4-VP	DVB	ACN:DMF (1.5:1, v/v)
NIP5	-	4-VP	EGDMA	Methanol

*The molar ratio of template molecule to monomer in MIPs is 1:10, and the molar ratio of monomer to cross-linker of MIPs and NIPs is 1:3*.

### Static Adsorption Experiments

Ten milligrams of ACT MIPs was accurately weighed and placed in a 10-ml black cap bottle. Ten milliliters of ACT standard solution with 0.50 mg/ml concentration was added. The black cap bottle was placed in a thermostatic shaker. The temperature was set at 30°C, the speed was 150 rpm, and the adsorption process was 24 h. One milliliter of the solution was used to determine the content of ACT in the filtrate by HPLC.

### Dynamic Adsorption Experiments

Fifty milliliters of sample solution (standard solution or *C. tubulosa* extract) was placed in a cap bottle, and 20.00 mg MIPs was added and placed in a shaker at 30°C for 24 h. The samples were sampled at a set time within 24 h. Compared with SPE, d-SPE was more valuable, because the process of d-SPE can avoid the problems on variations of pressure and flow rate. One milliliter of solution was taken from each sample and the concentration of ACT in the solution was determined by HPLC.

In order to investigate the adsorption process, the pseudo-first-order reaction model equation and pseudo-second-order reaction model equation were used to describe the adsorption process of ACT on adsorbents. The pseudo-first-order reaction model equation was as follows:

(1) $$\begin{array}{r} \log\left(q_{e}-q_{t}\right)=\log q_{e}-\frac{K_{1}}{2.303}t \end{array}$$

where *K*_1_ is the adsorption rate constant of pseudo-first-order kinetic model; *t* (min) is time; *q*_*t*_ is the adsorption capacity of time *t*.

The quasi-second-order reaction model equation is as follows:

(2) $$\begin{array}{r} \frac{t}{q_{t}}=\frac{1}{K_{2}q_{e}^{2}}+\frac{1}{q_{e}} \end{array}$$

where *K*_2_ is the adsorption rate constant of the pseudo-second-order kinetic model.

### Selectivity Experiments

The standard solutions of ACT and ECH with a concentration of 0.50 mg/ml were put into the black cap bottle for adsorption experiments, such as the static adsorption conditions above. One milliliter was extracted from the adsorbed solution, and the ACT and ECH content in the filtrate were determined by HPLC.

The adsorption capacity *Q* (mg/g) for the template bound to MIPs was calculated according to the following equation (Zhao et al., 2017):

(3) $$\begin{array}{r} Q=\frac{\left(C_{0}-C_{1}\right)}{m}V \end{array}$$

where *C*_0_ (mg/ml) and *C*_1_ (mg/ml) are the initial concentration and equilibrium concentration of standard solutions (Wang H.B. et al., 2019), *V* (ml) is the volume of standard solution, and *m* (g) is the weight of the MIP.

The adsorption selectivity of MIPs was evaluated by tow parameters such as the imprinting factor (*IF*) and adsorption separation factor (α).

The calculation of the imprinting factor is as follows:

(4) $$\begin{array}{r} IF=\frac{Q_{MIP}}{Q_{NIP}} \end{array}$$

where *Q*_*MIP*_ and *Q*_*NIP*_ are the adsorption capacity *Q*_*e*_ (mg/g) of the bound analyte at equilibrium on the MIP and the NIP, respectively.

The calculation of the adsorption separation factor is as follows:

(5) $$\begin{array}{r} \alpha =\frac{K_{D,template}}{K_{D,analog}} \end{array}$$

(6) $$\begin{array}{r} K_{D}=\frac{\left(C_{0}-C_{e}\right)V}{C_{e}m} \end{array}$$

where *C*_*e*_ (mg/ml) is the concentration of the solution after absorbed, *C*_0_ (mg/ml) is the initial concentration of the solution, *V* (ml) is the volume of the solution in the absorbed process, and *m* (g) is the mass of the sorbent. *K*_*D, template*_ and *K*_*D, analog*_ are the static distribution coefficients toward the template molecules and analog, respectively (Singh et al., 2013).

## Results and Discussion

### Characterization of MIPs

**Figure 1:**
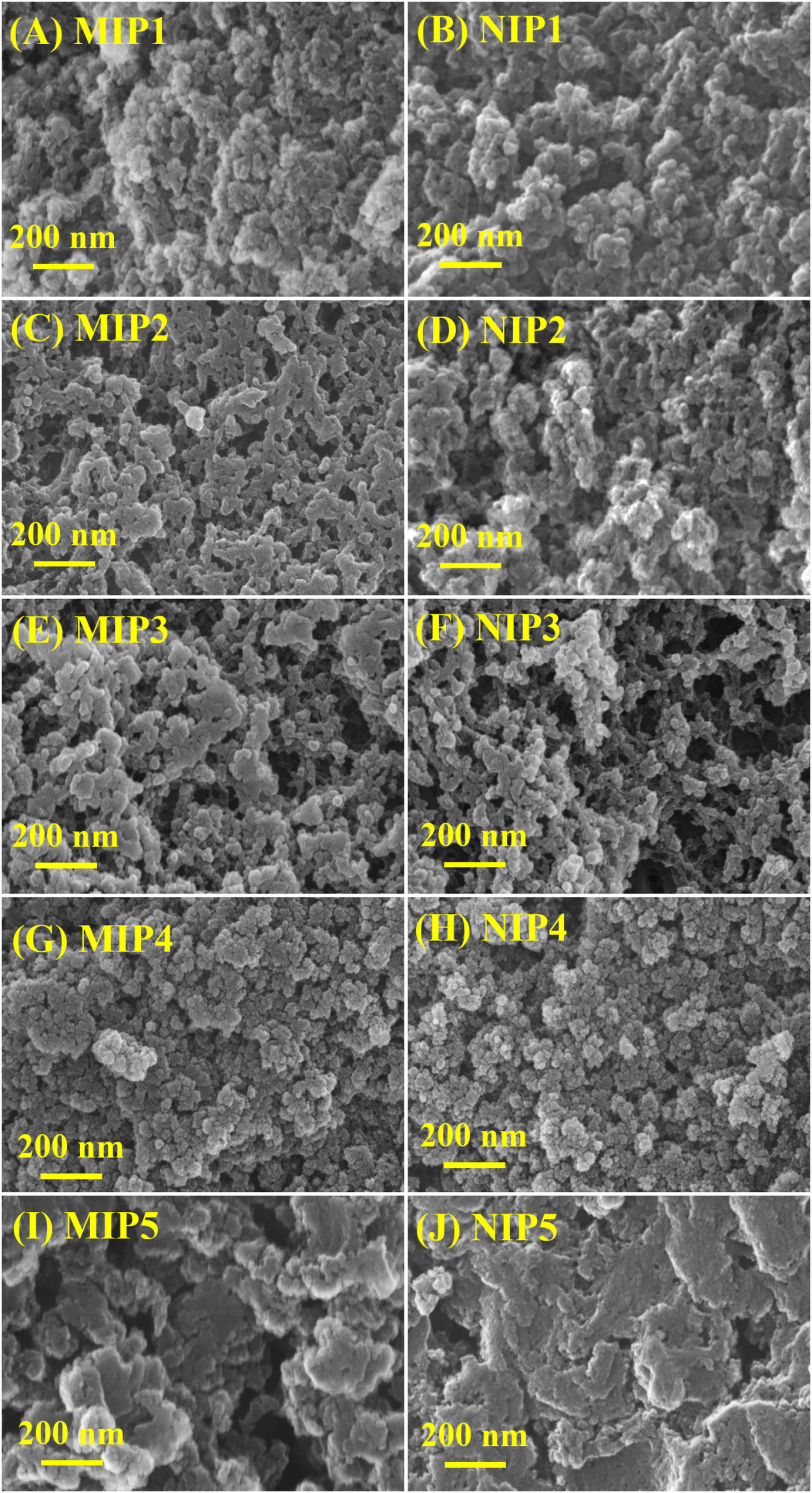
SEM of **(A)** MIP1, **(B)** NIP1, **(C)** MIP2, **(D)** NIP2, **(E)** MIP3, **(F)** NIP3, **(G)** MIP4, **(H)** NIP4, **(I)** MIP5, and **(J)** NIP5.

The SEM images of MIPs and NIPs are shown in Figure 1. As can be seen from Figure 1, MIPs and NIPs show different loose structures in different monomers, cross-linking agents, and solvents. MIP1–MIP4 and NIP1–NIP4 are heterogeneous particles with irregular shape and different size. MIP5 and NIP5 are heterogeneous lamellar with irregular shape and different size. The structure of MIP1 prepared in EGDMA is looser than that MIP4 prepared in DVB (Figures 1A,G). The structure of MIP1 with ACN and DMF as solvent (1:1.5, v/v) is looser than that of MIP5 with methanol as solvent (Figures 1A,I). The loose structure can speed up the molecular mass transfer and improve the binding speed of MIPs and ACT.

**Figure 2:**
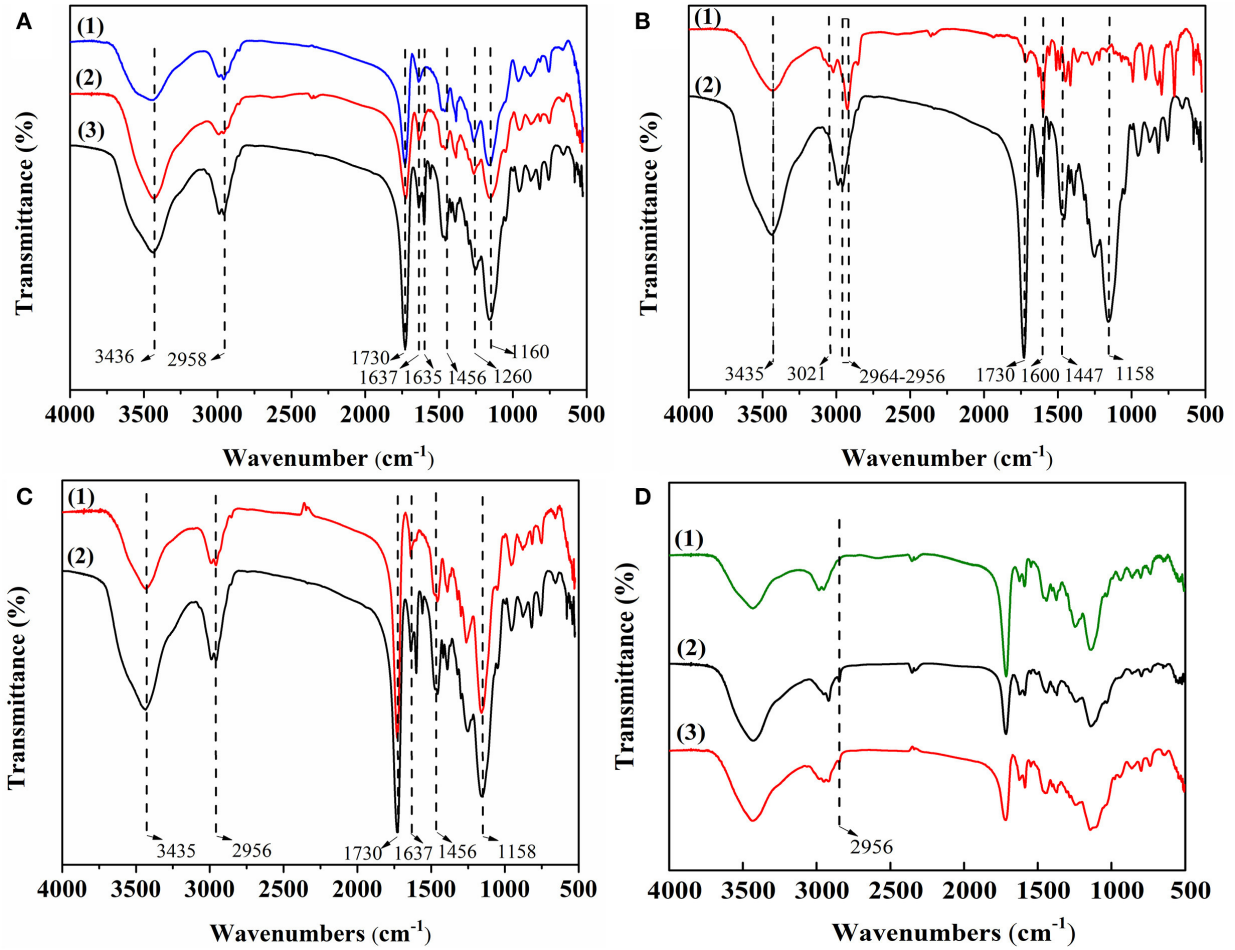
**(A)** FT-IR spectra of MIPs with different functional monomers: (1) 4-VP of MIP1; (2) MAA of MIP2, and (3) HEMA of MIP3; **(B)** FT-IR spectra of MIPs with different cross-linkers: (1) DVB of MIP4 and (2) EGDMA of MIP1; **(C)** FT-IR spectra of MIPs with different solvents: (1) MIP5 and (2) MIP1; **(D)** FT-IR spectra of (1) NIP1, (2) MIP1-ACT, and (3) MIP1.

Figure 2 shows FT-IR spectra of MIPs with different functional monomers (A), cross-linkers (B), and solvents (C). As shown in Figure 2A, the C = N stretching vibration peak and the C = C stretching vibration peak appeared in 4-VP at 1,637 cm^−1^ and 1,456 cm^−1^, respectively (Figure 2A, trace1). The C = O stretching vibration peak and the C-O stretching vibration peak appeared in MAA at 1,730 and 1,260 cm^−1^, respectively (Figure 2A, trace2). The stretching vibration peak of β-hydroxy and the stretching vibration peak of methylene appeared in HEMA at 3,436 and 2,958 cm^−1^, respectively (Figure 2A, trace3). The results indicate that 4-VP, MAA, and HEMA were successfully polymerized into the MIPs. As shown in Figure 2B, the stretching vibration absorption peaks of unsaturated C–H bond and skeleton vibration absorption peaks of benzene ring appeared in DVB at 3,021 and 1,600 cm^−1^, respectively (Figure 2B, trace1). The stretching vibration absorption peaks of C = O and the stretching vibration absorption peaks of O-C-O appeared in EGDMA at 1,730 and 1,160 cm^−1^, respectively (Figure 2B, trace2). The results indicate that MIP4 and MIP1 were successfully polymerized. As shown in Figure 2C, it can be seen that MIP1 and MIP5 had no peculiar characteristic peak, indicating its chemical structure is similar.

Figure 2D is the FT-IR spectra of NIP1, MIP1-ACT, and MIP1. The infrared spectrum of the NIP1 and the infrared spectrum of the MIP1 have the same characteristic peak, indicating that its chemical structure is similar (Figure 2D, trace1 and trace3). The infrared spectrum of the MIP1-ACT produced a new peak at 2,956 cm^−1^ compared to the infrared spectrum of the MIP1 (Figure 2D, trace2 and trace3), due to the acyl group of ACT that interacts with 4-VP to form a hydrogen bond association on the MIP1, indicating that the template molecule has been adsorbed to the MIP1 through hydrogen bonding.

**Figure 3:**
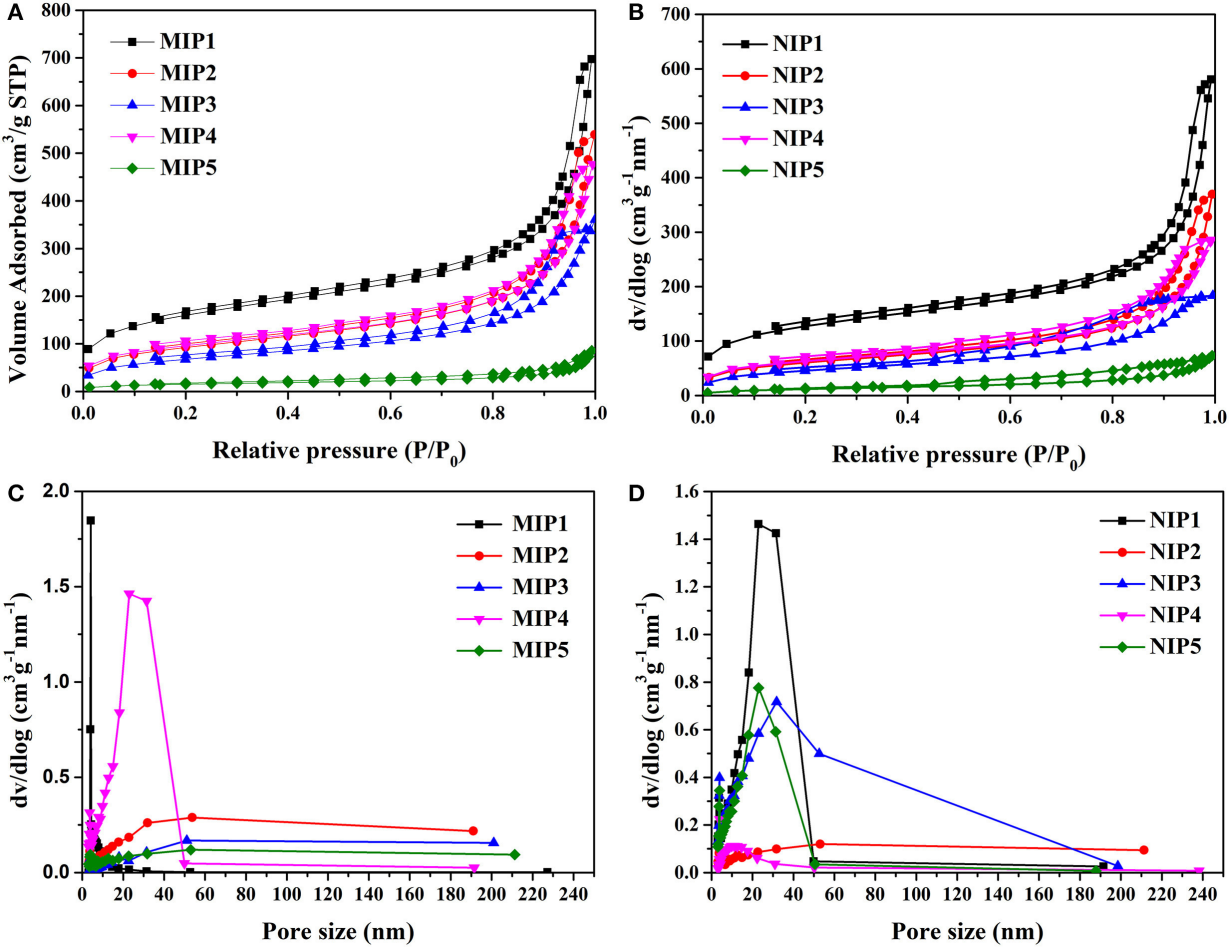
BET data **(A)** hysteresis curve of MIPs, **(B)** hysteresis curve of NIPs, **(C)** pore distribution diagram of MIPs, and **(D)** pore distribution diagram of NIPs.

The hysteretic curve and pore size distributions of MIPs and NIPs are shown in Figure 3. The hysteretic curves of MIPs and NIPs exhibited “type IV” isotherm (Figures 3A,B). The pore size distributions of MIPs and NIPs were distributed in the range of 5–50 nm, which indicated that the pores of MIPs and NIPs belonged to mesopores (Figures 3C,D).

The data of specific surface area, pore volume, and pore size for MIPs and NIPs are listed in Table 4. The specific surface area of MIP1 was 593.91 m^2^/g with a pore size of 10.91 nm. The specific surface area of NIP1 was 427.12 m^2^/g with a pore size of 7.94 nm. Obviously, the specific surface area and pore size of the MIP1 were larger than that of the NIP1. This can be attributed to the presence of imprinted holes on the surface of MIP1. The high specific surface area and large pore size of MIP1 are favorable for increasing the adsorption capacity of MIP1 for ACT.

**Table 4:** BET data for MIPs and NIPs.

**Polymers**	**Hysteresis curve**	**Pore size (nm)**	**Pore volume (cm^**3**^/g)**	**Surface area (m^**2/**^g)**
MIP1	H4	10.91	1.08	539.91
MIP2	H4	10.01	0.81	317.76
MIP3	H4	9.09	0.54	232.76
MIP4	H4	8.91	0.74	327.49
MIP5	H4	10.06	0.13	51.88
NIP1	H4	8.41	0.89	427.12
NIP2	H4	7.94	0.57	206.95
NIP3	H4	7.11	0.28	159.88
NIP4	H4	8.12	0.44	215.24
NIP5	H4	10.29	0.11	41.83

**Figure 4:**
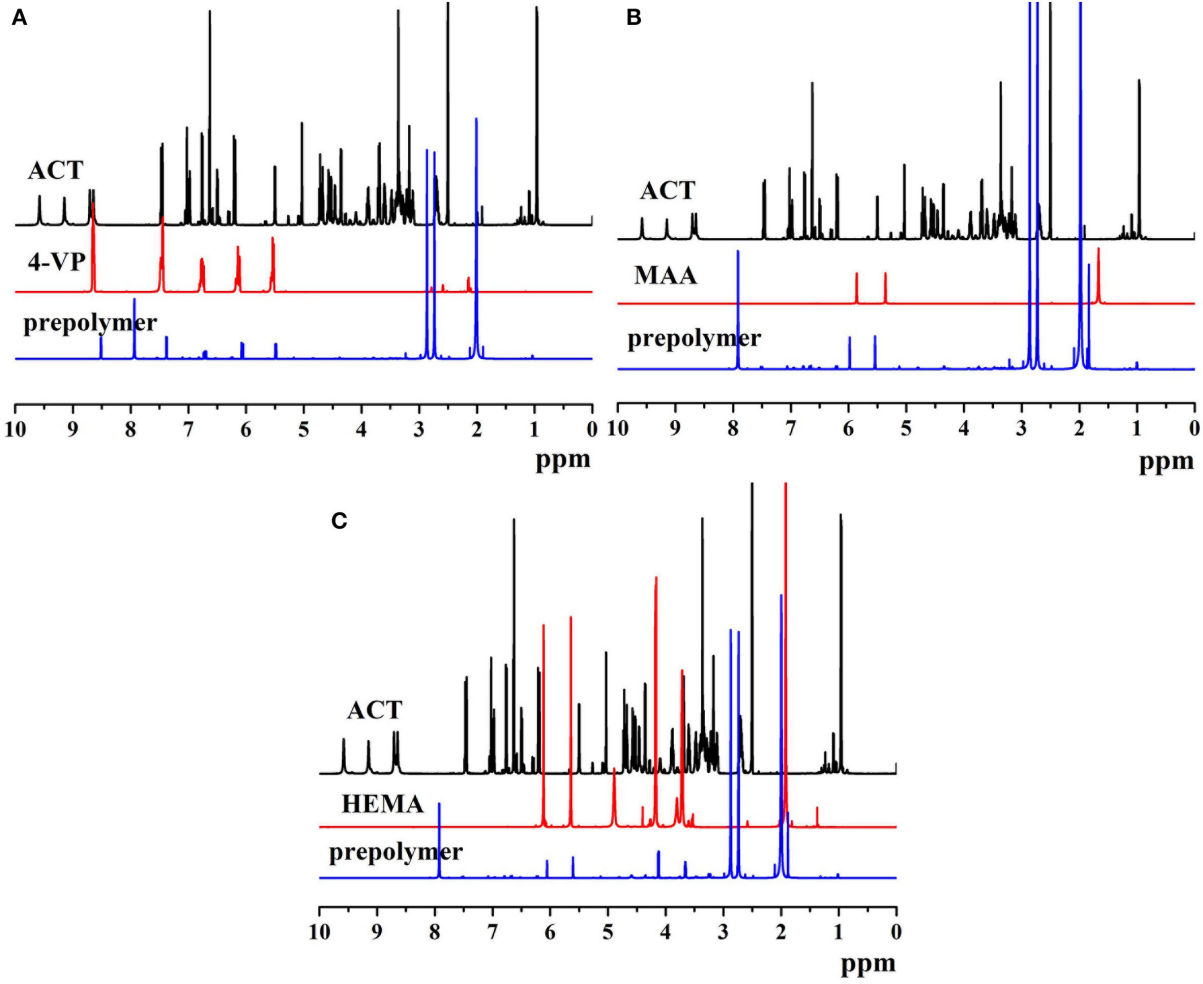
The ^1^H NMR spectra of **(A)** ACT, 4-VP, and ACT-4-VP prepolymer, **(B)** ACT, MAA, and ACT-MAA prepolymer, and **(C)** ACT, HEMA, and ACT-HEMA prepolymer.

The ^1^H NMR spectra of ACT, monomers, and prepolymers are shown in Figure 4. The proton peaks of different phenolic hydroxyl groups on ACT appeared at 7.50, 6.75, 6.20, 5.02, 4.35, and 3.52 ppm (Figures 4A–C). As shown in Figure 4A, the proton peaks on the pyridine groups of 4-VP exhibited at 8.64, 7.45, 6.75, 6.17, and 5.54 ppm. Compared with the proton peaks of ACT and 4-VP, prepolymer of MIP1 appeared new proton peaks at 8.52, 8.01, 7.49, 6.67, 6.13, and 5.54 ppm, which resulted from the formed hydrogen bonds between ACT and 4-VP. As shown in Figure 4B, the proton peaks of MAA exhibited at 5.94, 5.53, and 1.47 ppm. Compared with the proton peaks of ACT and MAA, prepolymer of MIP2 presented new proton peaks at 7.95, 5.98, 5.67, and 1.03 ppm, which resulted from the formed hydrogen bonds between ACT and MAA. As shown in Figure 4C, the proton peaks of HEMA exhibited at 6.04, 5.56, 4.85, 4.11, and 3.75 ppm. Compared with the proton peaks of ACT and HEMAA, prepolymer of MIP3 exhibited new proton peaks at 7.95, 6.15, 5.76, 4.32, and 3.66 ppm, which resulted from the formed hydrogen bonds between ACT and HEMA.

### Adsorption Experiment

In the process of MIPs preparation, the appropriate functional monomers determine whether molecularly imprinted polymers have excellent recognition ability. This is because different functional monomers contain different functional groups and the interaction between template molecules is different. According to the acidity and alkalinity, functional monomers can be further divided into acidic functional monomers, basic functional monomers, and neutral functional monomers, and acidic template molecules should be selected as basic functional monomers. Hydrogen bonds can be formed between different functional groups of monomers and hydroxyl groups of the template molecules in MIPs (Hammam et al., 2018; Panjan et al., 2018). 4-VP contains pyridine groups, and the pyridine groups can form hydrogen bonds with the hydroxyl groups in the template molecule of ACT. MAA contains carboxyl groups, and carboxyl groups can form hydrogen bonds with the hydroxyl groups in the template molecule of ACT. HEMA contains hydroxyl groups, and hydroxyl groups can form hydrogen bonds with template molecules of ACT (Yesilova et al., 2018; Haginaka et al., 2019; Luo et al., 2019). The static adsorption data of MIPs and NIPs are listed in Table 5. According to the data listed in Table 5, MIP1 has the highest adsorption capacity and the highest imprinting factor. The adsorption capacity of MIP1 was 168.05 mg/g, and the imprinting factor was 2.69. On one hand, MIP1 had large amounts of imprinted holes, which can match the spatial configuration of ACT, achieving selective adsorption for ACT. On the other hand, the hydroxyl groups on ACT contacted with the N–H groups of MIP1 in the adsorption process to form hydrogen bonds, which can increase adsorption capacity of ACT.

**Table 5:** Adsorption data of MIPs and NIPs.

**Polymers**	***Q*_**ACT**_ (mg/g)**	**IF**
MIP1	168.05 ± 4.65	2.69 ± 0.13
MIP2	113.58 ± 5.64	2.42 ± 0.15
MIP3	124.01 ± 4.39	0.87 ± 0.008
MIP4	124.59 ± 5.64	0.84 ± 0.005
MIP5	5.79 ± 6.34	0.32 ± 0.21
NIP1	62.58 ± 4.85	-
NIP2	46.91 ± 5.76	-
NIP3	143.13 ± 5.83	-
NIP4	147.77 ± 6.32	-
NIP5	17.96 ± 5.34	-

*-, not available for data (n = 3)*.

**Figure 5:**
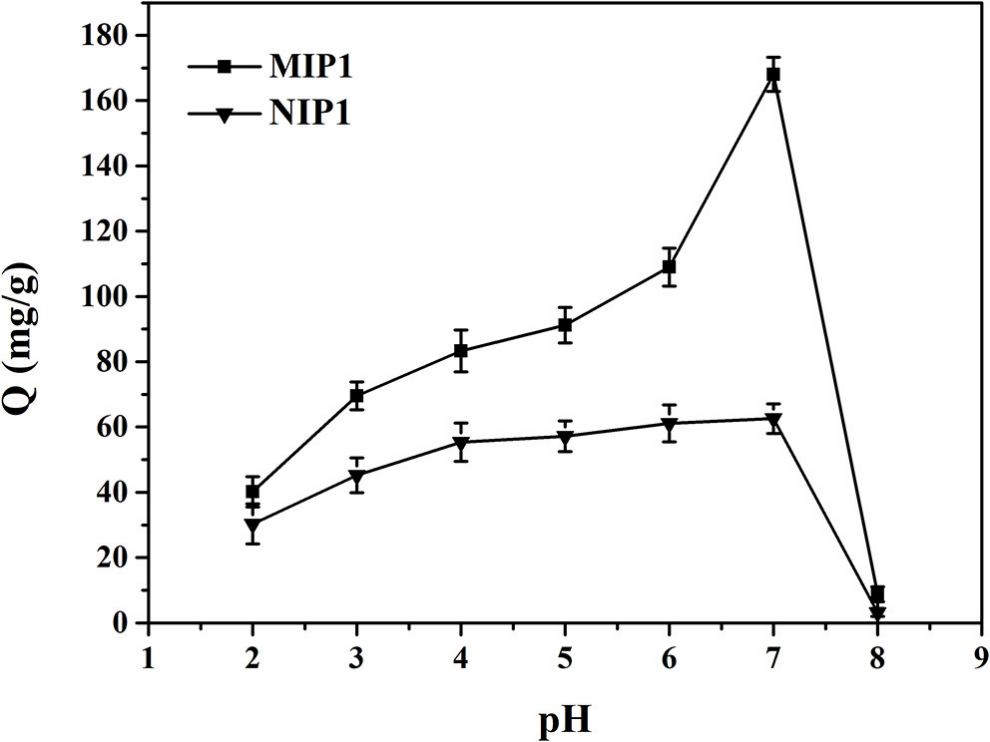
The effect of pH on the adsorption experiments of MIP1 and NIP1 for ACT.

The effect of pH on the adsorption performance of MIP1 and NIP1 for ACT is shown in Figure 5. It can be seen from Figure 5 that the optimal pH value was 7 for adsorption of ACT by MIP1 and NIP1. The reasons are as follows: ACT had a large number of phenolic hydroxyl groups and belonged to the weak acidic molecules, and the different pH values affected the stability of ACT. The stability of phenolic hydroxyl groups on ACT molecules would decrease at pH>7 and pH <7. This will reduce the amount of ACT adsorbed by MIP1 and NIP1. Thus, the optimum pH was 7 for MIP1 and NIP1 adsorption.

### Isothermal Adsorption Experiment

**Figure 6:**
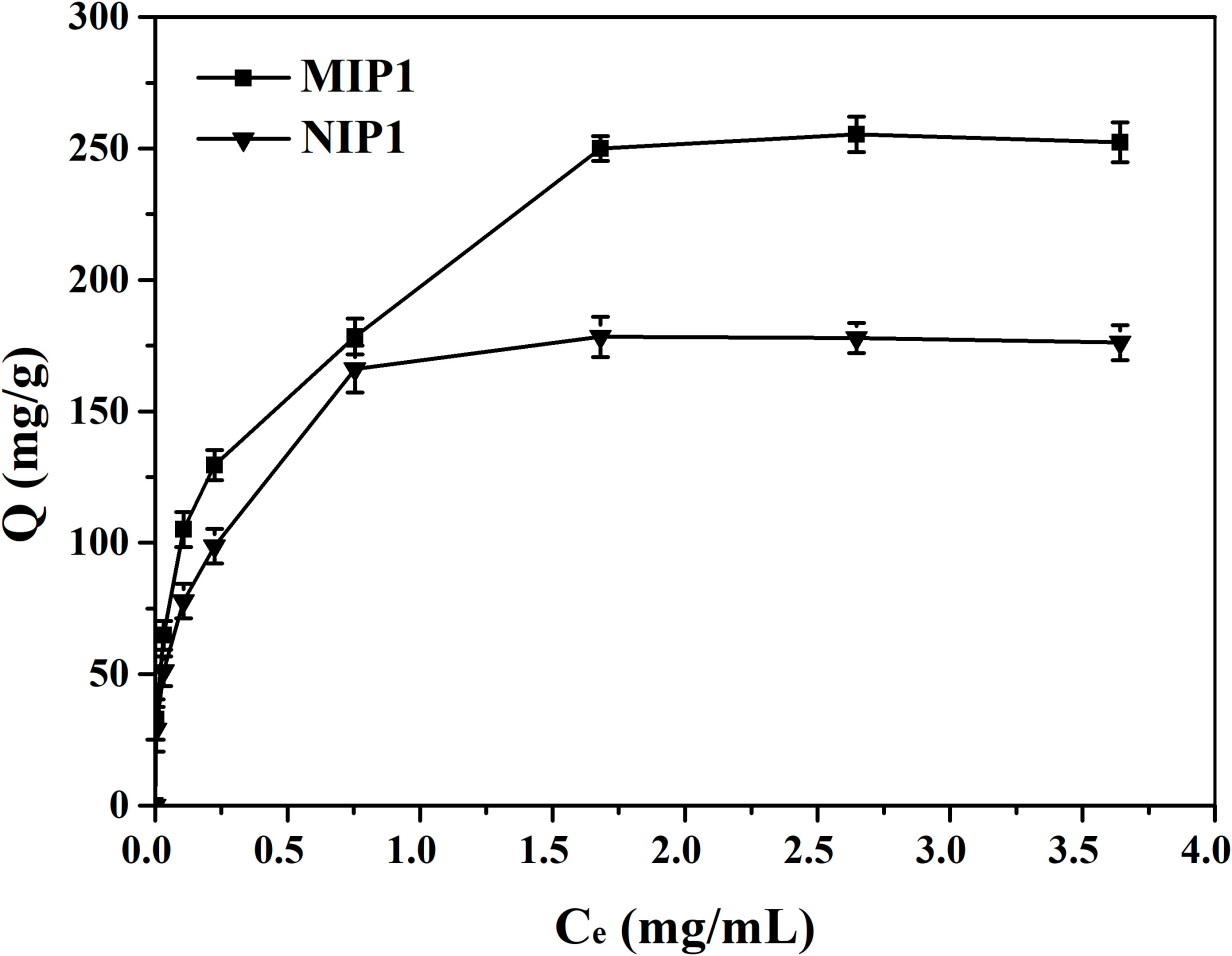
Adsorption isotherms of MIP1 and NIP1 for ACT.

The isotherm adsorption curves of MIP1 and NIP1 for ACT were shown in Figure 6. It can be seen that the adsorption capacity of MIP1 for ACT increases with the increase of the initial ACT concentration, and this might be the reason that the amount of ACT was not enough to saturate the specific binding cavities. The adsorption curve reached the saturation and tended to be stable when the initial concentration exceeded 1.50 mg/ml, and the maximum adsorption capacity of MIP1 was 250.00 mg/g, which indicated that a great many ACT specific binding sites were produced during imprinting process. Figure 4 also shows that the amounts of ACT bound to the MIPs were in a high level compared with those of the NIPs under the same conditions.

### Scatchard Plot Analysis

**Figure 7:**
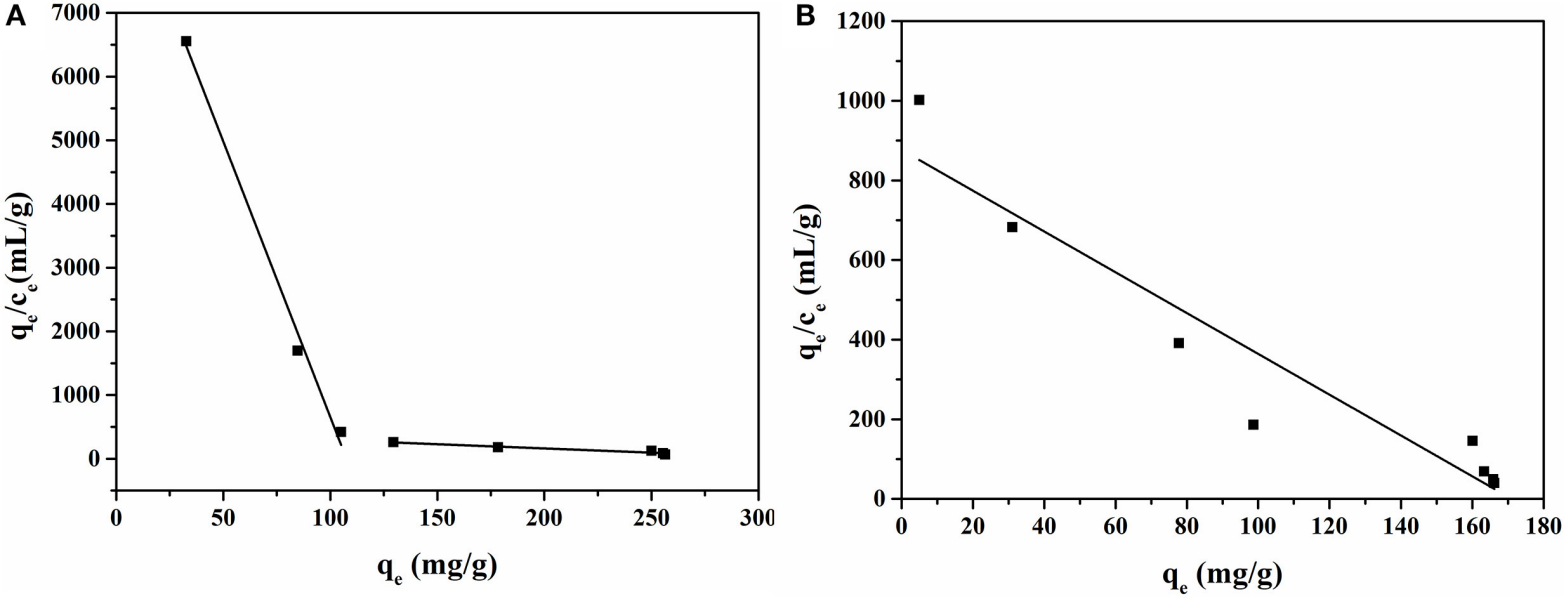
Scatchard plot of MIP1 **(A)** and NIP1 **(B)** for ACT.

The Scatchard plot of MIP1 and NIP1 for ACT is shown in Figure 7. The binding properties of MIPs were determined by Scatchard plot analysis, which was based on the following equation:

(7) $$\begin{array}{r} \frac{q_{e}}{c_{e}}=\frac{B_{\max}-q_{e}}{K_{a}} \end{array}$$

where *c*_e_ is the equilibrium concentration of ACT in the solution, *q*_e_ is the amount of ACT bound to the MIPs at equilibrium, *B*_max_ is the apparent maximum binding amount, and *K*_a_ is the dissociation constant.

The Scatchard plot of MIP1 contained two different linear regression lines, suggesting two types of binding sites. As shown in Figure 7A, the left line suggested that the MIP1 had high binding affinity with ACT in the concentration range of 0.005–0.25 mg/ml. The *K*_a_ and *B*_max_ were found to be 1.94 mg/ml and 18,080.15 mg/g for dry polymer, respectively, and they were calculated from the intercept and slope of the regression equation *q*_e_/*c*_e_ = −86.54*q*_e_ + 9310.66 (*R*^2^ = 0.98). The right line indicated that MIP1 had low binding affinity in the concentration range of 0.25–4.00 mg/ml. The *K*_a_ and *B*_max_ were found to be 127.31 mg/ml and 54,316.81 mg/g for dry polymer, respectively, and they were calculated from the regression equation *q*_e_/*c*_e_ = −1.32*q*_e_ + 426.65 (*R*^2^ = 0.91). Meanwhile, it can be seen from the two equations that the slope of the straight line on the left side was small, and the slope of the straight line on the right side was large. The small slope had high binding affinity with ACT for MIP1.

The Scatchard plot of NIP1 shows a straight line, indicating that there is only one type of binding site in NIP1. The *K*_a_ and *B*_max_ were found to be 12.19 mg/ml and 10,689.41 mg/g for dry polymer, respectively, and they were calculated from the regression equation *q*_*e*_/*c*_*e*_ = −5.13*q*_*e*_+ 876.91 (*R*^2^ = 0.89).

### Adsorption Kinetics Study

**Figure 8:**
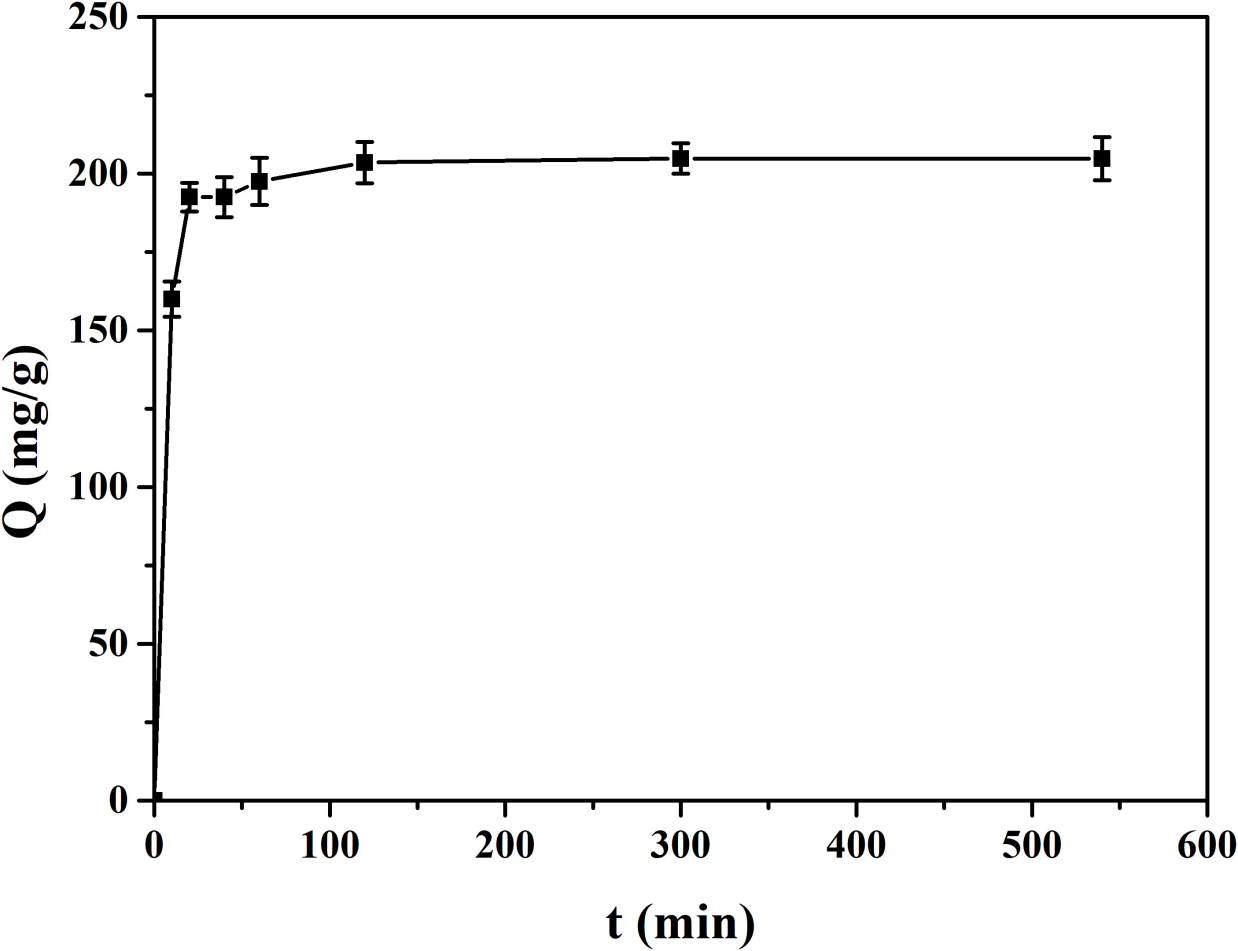
Adsorption kinetics of MIP1 on ACT.

Figure 8 shows the adsorption kinetics curve of MIP1 for ACT. The dynamic adsorption experiments were carried out in ACT solution with an initial concentration of 0.50 mg/ml; it can be seen that the adsorption capacity of MIP1 for ACT increases rapidly in 20 min and slowly in 100 min, but it does not change much after 150 min. Therefore, the equilibrium adsorption time of MIP1 is 150 min and the equilibrium adsorption capacity is 204.08 mg/g. At the beginning of dynamic adsorption, there are more free ACT molecules in ACT solution and more specific recognition sites in MIP1, so the hydrogen bonding rate between MIP1 and ACT is fast. After 20 min, the number of specific recognition sites of MIP1 and the number of free radicals in the ACT solution decreased, which reduced the binding rate of MIP1 and ACT, eventually reaching dynamic equilibrium.

**Figure 9:**
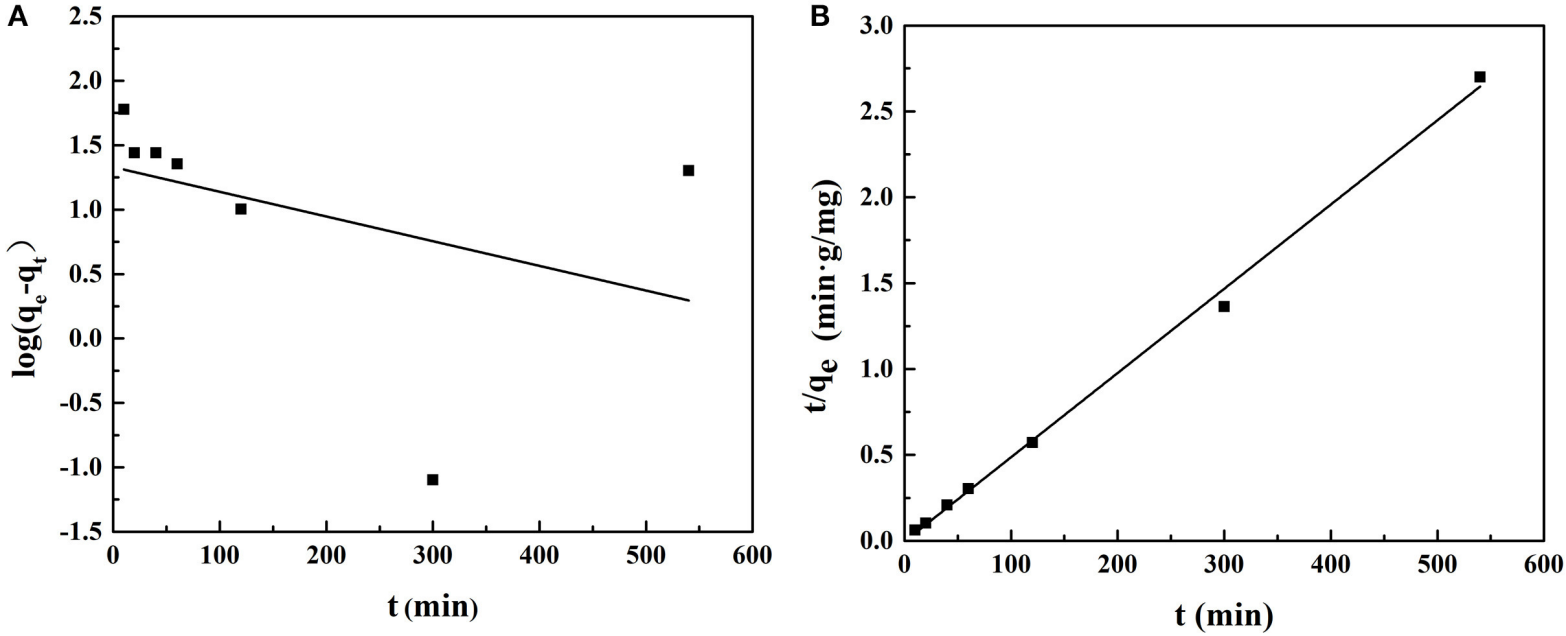
Adsorption dynamic curves of ACT with the fitting to **(A)** the pseudo-first-order model and **(B)** the pseudo-second-order model.

Figure 9 shows the quasi-first-order kinetic model and quasi-second-order kinetic model of ACT adsorption on MIP1, and Table 6 shows the data fitted by the kinetic model. The equilibrium adsorption capacity in the dynamic adsorption equilibrium experiment of MIP1 was 204.08 mg/g. The experimental data are consistent with the pseudo-second-order kinetic fitting data, which proves that the adsorption behavior of MIP1 conforms to the pseudo-second-order kinetic model (*R*^2^ > 0.99). The dynamic adsorption equilibrium accords with the quasi-second-order kinetics; it indicates that chemical adsorption is a speed-control step in the adsorption process. Therefore, the adsorption behavior of ACT on MIP1 may be hydrogen bonding.

**Table 6:** Fitting of quasi-first-order and quasi-second-order dynamic models.

**Adsorbent**	**Quasi-first-order kinetic fit curve**			**Quasi-second-order dynamic fitting curve**		
	***K*_**1**_**	***Q*_**e**_ (mg/g)**	***R*^**2**^**	***K*_**2**_**	***Q*_**e**_ (mg/g)**	***R*^**2**^**
MIP1	0.0044	21.39	0.15	0.0052	204.08	0.99

### Purification of ACT From *C. tubulosa*

#### Preparation of the Extracts of *C. tubulosa*

Twenty grams of *C. tubulosa* powder was dispersed in a 50% ethanol solution at 70°C. The extraction was carried out for 2 min under a high shear homogenizer at 16,000 rpm. The extracts were filtered through a 0.22-μm filter to obtain the extract of *C. tubulosa*.

#### Preparation of Solid Phase Extraction Column and Solid Phase Extraction of ACT in the Extracts of *C. tubulosa*

One hundred milligrams of MIP1 was dispersed in the 50% ethanol solution and loaded into a solid phase extraction column, and the SPE column of MIP1 was then rinsed with a 50% ethanol solution at a flow rate of 2.00 ml/min for 10 min. The extracts of *C. tubulosa* were injected into the SPE column at a flow rate of 2.00 ml/min, and the sample concentration of the effluent was measured. The eluate was collected after the SPE column of MIP1 was eluted with 90% ethanol solutions and 10% ethanol solutions, respectively (Gao et al., 2016). The collected eluent was dried and dissolved in water for constant volume, and then the content of ACT was measured by HPLC.

**Figure 10:**
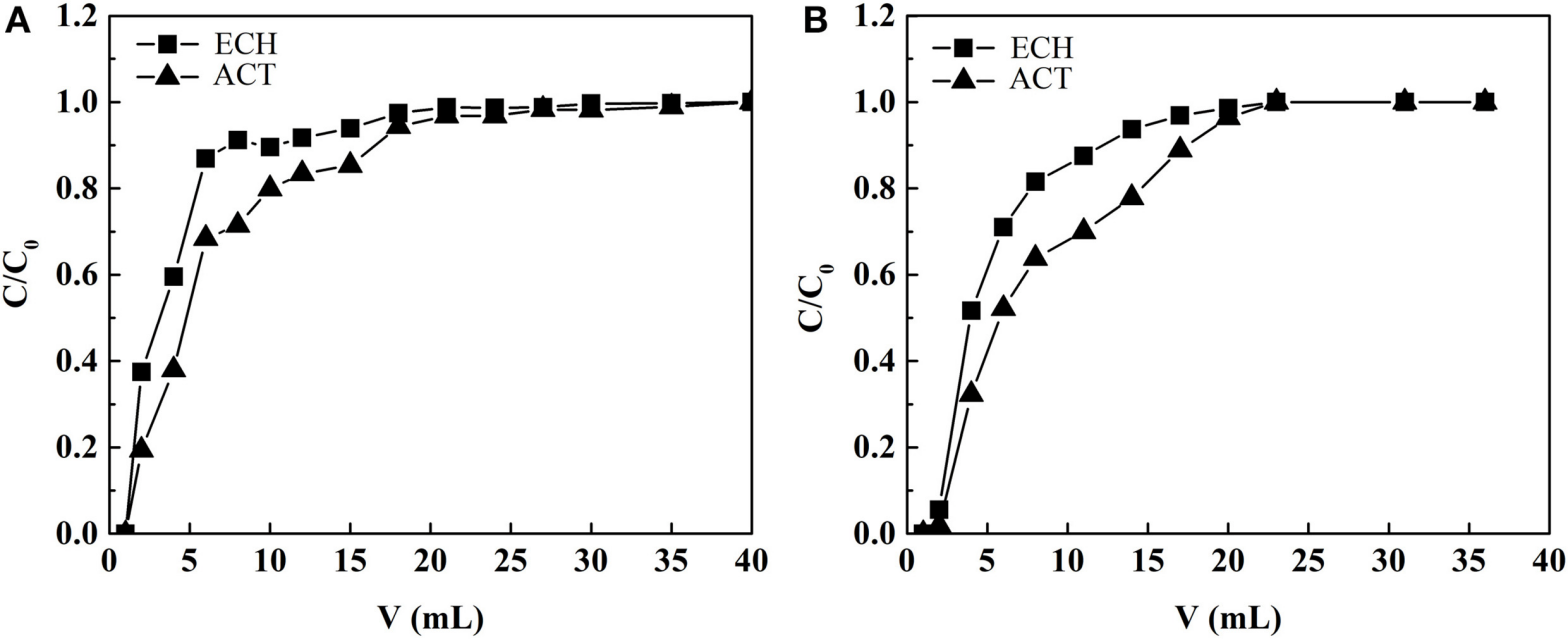
The relationship between effluent volume and effluent concentration in **(A)** MIP1 and **(B)** NIP1 adsorption process.

Figure 10 shows the relationship between effluent volume and effluent concentration in the adsorption process of MIP1 and NIP1, respectively. The contents of ECH and ACT in the extract of *C. tubulosa* were 2.44 and 0.53 mg/ml, respectively. As can be seen from Figure 10, when the effluent volume is 40.00 ml, the concentrations of ACT and ECH in the effluent are the same as those in the extract of *C. tubulosa*, indicating that MIP1 reaches the adsorption equilibrium. When the effluent volume was 23.00 ml, the concentrations of ACT and ECH in the effluent were the same as those in the extract of *C. tubulosa*, indicating that NIP1 reached the adsorption equilibrium. The binding amount of ACT by MIP1 solid phase extraction column is larger than that by NIP1 solid phase extraction column; it indicated that MIP1 has excellent imprinting effect on ACT.

The content of ECH in the extract of *C. tubulosa* was 4.58 times that of ACT. The adsorption capacity of MIP1 on ACT and ECH was 36.86 and 88.67 mg/g, respectively. After the eluent is eluted, the recovery rate of ACT was 90.09%. The purity of ACT increased from 1.99 to 27.88%, and the increasing amplitude of purity is 1301.00%, which was higher than the increment of 960.00% by adsorption of microporous resins (HPD300) (Liu et al., 2013). The increase in purity of ACT results from the selective adsorption of ACT by molecularly imprinted cavity.

## Conclusions

An imprinting material with a high selective adsorption capacity is used for simple and rapid separation of ACT. MIPs were investigated in terms of static adsorption experiments, dynamic adsorption experiments, and selectivity experiments. The experimental results showed that MIP1 exhibited the optimal adsorption performance to ACT. MIP1 was prepared with ACT as template molecule, 4-VP as a functional monomer, EGDMA as a cross-linking agent, the volume ratio of ACN and DMF of 1:1.5 (v/v) as a solvent, and AIBN as initiator. The adsorption results displayed that the adsorption capacity of MIP1 to ACT reached 112.60 mg/g, and the separation factor of ACT/ECH was 4.68. The dynamic adsorption of ACT accorded with the quasi-second-order kinetics; it indicated that the adsorption process of MIP1 is the process of chemical adsorption to ACT. MIPs with high selectivity make it a potential adsorption material for the purification of plant active ingredients.

## Data Availability Statement

All datasets generated for this study are included in the article/supplementary material.

## Author Contributions

XZ carried out experiments and wrote the manuscript. WP designed the experiments, provided useful suggestions, and solved the problems in the experiments. RG provided the experiment tools. XL contributed to the study design, manuscript revision, and final version.

### Conflict of Interest

The authors declare that the research was conducted in the absence of any commercial or financial relationships that could be construed as a potential conflict of interest.

## References

[B1] Ansari S. Karimi M. (2017). Novel developments and trends of analytical methods for drug analysis in biological and environmental samples by molecularly imprinted polymers. Trends Anal. Chem. 89, 146–162. 10.1016/j.trac.2017.02.002

[B2] BelBruno J. J. (2019). Molecularly imprinted polymers. Chem. Rev. 119, 94–119. 10.1021/acs.chemrev.8b00171 30246529

[B3] Cantarella M. Carroccio S. C. Dattilo S. Avolio R. Castaldo R. Puglisi C. (2019). Molecularly imprinted polymer for selective adsorption of diclofenac from contaminated water. Chem. Eng. J. 367, 180–188. 10.1016/j.cej.2019.02.146

[B4] Cowen T. Karim K. Piletsky S. (2016). Computational approaches in the design of synthetic receptors - A review. Anal. Chim. Acta 936, 62–74. 10.1016/j.aca.2016.07.027 27566340

[B5] Diltemiz S. E. Kecili R. Ersoez A. Say R. (2017). Molecular imprinting technology in quartz crystal microbalance (QCM) sensors. Sensors 17, 454–454. 10.3390/s17030454 PMC5375740 28245588

[B6] Dong B. Yuan X. Zhao Q. Feng Q. Liu B. Guo Y.. (2015). Ultrasound-assisted aqueous two-phase extraction of phenylethanoid glycosides from Cistanche deserticola Y. C. Ma stems. J. Sep. Sci. 38, 1194–1203. 10.1002/jssc.201401410 25604674

[B7] Fu C. Li J. Aipire A. Xia L. Yang Y. Chen Q.. (2018). *Cistanche tubulosa* phenylethanoid glycosides induce apoptosis in Eca-109 cells via the mitochondria-dependent pathway. Oncol. Lett. 17, 303–313. 10.3892/ol.2018.9635 30655768 PMC6313098

[B8] Gao D. Yang F. Xia Z. Zhang Q. (2016). Molecularly imprinted polymer for the selective extraction of luteolin from *Chrysanthemum morifolium* ramat. J. Separation Science. 39, 3002–3010. 10.1002/jssc.201600520 27288270

[B9] Giebultowicz J. Sobiech M. Ruzycka M. Lulinski P. (2019). Theoretical and experimental approach to hydrophilic interaction dispersive solid-phase extraction of 2-aminothiazoline-4-carboxylic acid from human post-mortem blood. J. Chromatogr. A 1587, 61–72. 10.1016/j.chroma.2018.12.028 30579638

[B10] Haginaka J. Nishimura K. Kimachi T. Inamoto K. Takemoto Y. Kobayashi Y. (2019). Retention and molecular-recognition mechanisms of molecularly imprinted polymers for promazine derivatives. Talanta 205:20149. 10.1016/j.talanta.2019.120149 31450460

[B11] Hammam M. A. Wagdy H. A. El Nashar R. M. (2018). Moxifloxacin hydrochloride electrochemical detection based on newly designed molecularly imprinted polymer. Sens. Actua. B-Chem. 275, 127–136. 10.1016/j.snb.2018.08.041

[B12] Han L. Ji L. Boakye-Yiadom M. Li W. Song X. Gao X. (2012). Preparative isolation and purification of four compounds from Cistanches deserticola Y.C. Ma by high-speed counter-current chromatography. Molecules 17, 8276–8284. 10.3390/molecules17078276 22781440 PMC6268931

[B13] Hong S. She Y. Cao X. Wang M. He Y. Zheng L.. (2019). A novel CdSe/ZnS quantum dots fluorescence assay based on molecularly imprinted sensitive membranes for determination of triazophos residues in cabbage and apple. Front. Chem. 7:130. 10.3389/fchem.2019.00130 30937301 PMC6432856

[B14] Hrobonova K. Machynakova A. Cizmarik J. (2018). Determination of dicoumarol in *Melilotus officinalis* L. by using molecularly imprinted polymer solid-phase extraction coupled with high performance liquid chromatography. J. Chromatogr. A 1539, 93–102. 10.1016/j.chroma.2018.01.043 29395163

[B15] Huang Y. Pan J. Liu Y. Wang M. Deng S. Xia Z. (2019). A SPE method with two MIPs in two steps for improving the selectivity of MIPs. Anal. Chem. 91, 8436–8442. 10.1021/acs.analchem.9b01453 31132265

[B16] Li C. Ma X. Zhang X. Wang R. Chen Y. Li Z. (2016). Magnetic molecularly imprinted polymer nanoparticles-based solid-phase extraction coupled with gas chromatography-mass spectrometry for selective determination of trace di-(2-ethylhexyl) phthalate in water samples. Anal. Bioanal. Chem. 408, 7857–7864. 10.1007/s00216-016-9889-x 27580604

[B17] Li F. Gao J. Li X. Li Y. He X. Chen L. (2019). Preparation of magnetic molecularly imprinted polymers functionalized carbon nanotubes for highly selective removal of aristolochic acid. J. Chromatogr. A 16, 168–177. 10.1016/j.chroma.2019.06.043 31303311

[B18] Li X. Cheng Y. Zhang H. Wang S. Jiang Z. Guo R.. (2015a). Efficient CO_2_ capture by functionalized graphene oxide nanosheets as fillers to fabricate multi-permselective mixed matrix membranes. ACS Appl. Mater. Interfaces. 7, 5528–5537. 10.1021/acsami.5b00106 25686296

[B19] Li X. Hou J. Guo R. Wang Z. Zhang J. (2019). Constructing unique cross-sectional structured mixed matrix membranes by incorporating ultrathin microporous nanosheets for efficient CO_2_ separation. ACS Appl. Mater. Interfaces 11, 24618–24626. 10.1021/acsami.9b07815 31257849

[B20] Li X. Ma L. Zhang H. Wang S. Jiang Z. Guo R. (2015b). Synergistic effect of combining carbon nanotubes and graphene oxide in mixed matrix membranes for efficient CO_2_ separation. J. Membr. Sci. 479, 1–10. 10.1016/j.memsci.2015.01.014

[B21] Li Z. Wang J. Chen X. Hu S. Gong T. Xian Q. (2019). A novel molecularly imprinted polymer-solid phase extraction method coupled with high performance liquid chromatography tandem mass spectrometry for the determination of nitrosamines in water and beverage samples. Food Chem. 292, 267–274. 10.1016/j.foodchem.2019.04.036 31054675

[B22] Liang R. Wang T. Zhang H. Yao R. Qin W. (2018). Soluble molecularly imprinted nanorods for homogeneous molecular recognition. Front. Chem. 6:81. 10.3389/fchem.2018.00081 29662877 PMC5890108

[B23] Liu B. Ouyang J. Yuan X. Wang L. Zhao B. (2013). Adsorption properties and preparative separation of phenylethanoid glycosides from Cistanche deserticola by use of macroporous resins. J. Chromatogr. B 937, 84–90. 10.1016/j.jchromb.2013.08.018 24022055

[B24] Lulinski P. (2017). Molecularly imprinted polymers based drug delivery devices: a way to application in modern pharmacotherapy. A review. Mater. Sci. Eng. 76, 1344–1353. 10.1016/j.msec.2017.02.138 28482502

[B25] Lulinski P. Bamburowicz-Klimkowska M. Dana M. Szutowski M. Maciejewska D. (2016). Efficient strategy for the selective determination of dopamine in human urine by molecularly imprinted solid-phase extraction. J. Separ. Sci. 39, 895–903. 10.1002/jssc.201501159 26732188

[B26] Lulinski P. Giebultowicz J. Wroczynski P. Maciejewska D. (2015). A highly selective molecularly imprinted sorbent for extraction of 2-aminothiazoline-4-carboxylic acid - Synthesis, characterization and application in post-mortem whole blood analysis. J. Chromatogr. A 1420, 16–25. 10.1016/j.chroma.2015.09.083 26463428

[B27] Luo Z. Xiao A. Chen G. Guo Q. Chang C. Zeng A. (2019). Preparation and application of molecularly imprinted polymers for the selective extraction of naringin and genistein from herbal medicines. Analy. Methods 11, 4890–4898. 10.1039/C9AY01503E

[B28] Ma X. Lin H. He Y. She Y. Wang M. Abd El-Aty A. M.. (2019). Magnetic molecularly imprinted polymers doped with graphene oxide for the selective recognition and extraction of four flavonoids from Rhododendron species. J. Chromatogr. A 1598, 39–48. 10.1016/j.chroma.2019.03.053 30940357

[B29] Marc M. Kupka T. Wieczorek P. P. Namiesnik J. (2018). Computational modeling of molecularly imprinted polymers as a green approach to the development of novel analytical sorbents. Trends Anal. Chem. 98, 64–78. 10.1016/j.trac.2017.10.020

[B30] Morikawa T. Xie H. Pan Y. Ninomiya K. Yuan D. Jia X.. (2019). A review of biologically active natural products from a desert plant *Cistanche tubulosa*. Chem. Pharm. Bull. 67, 675–689. 10.1248/cpb.c19-00008 31257323

[B31] Panjan P. Monasterio R. P. Carrasco-Pancorbo A. Fernandez-Gutierrez A. Sesay A. M. Fernandez-Sanchez J. F. (2018). Development of a folic acid molecularly imprinted polymer and its evaluation as a sorbent for dispersive solid-phase extraction by liquid chromatography coupled to mass spectrometry. J. Chromatogr. A 1576, 26–33. 10.1016/j.chroma.2018.09.037 30253912

[B32] Pei W. Guo R. Zhang J. Li X. (2019). Extraction of phenylethanoid glycosides from *Cistanche tubulosa* by high-speed shearing homogenization extraction. J. AOAC. Int. 102, 63–68. 10.5740/jaoacint.18-0039 30029698

[B33] Phungpanya C. Chaipuang A. Machan T. Watla-iad K. Thongpoon C. Suwantong O. (2018). Synthesis of prednisolone molecularly imprinted polymer nanoparticles by precipitation polymerization. Polym. Adv. Technol. 29, 3075–3084. 10.1002/pat.4428

[B34] Si Z. Yu P. Dong Y. Lu Y. Ta Z. Yu X.. (2019). Thermo-responsive molecularly imprinted hydrogels for selective adsorption and controlled release of phenol from aqueous solution. Front. Chem. 6:674. 10.3389/fchem.2018.00674 30740393 PMC6357936

[B35] Singh M. Kumar A. Tarannum N. (2013). Water-compatible 'aspartame'-imprinted polymer grafted on silica surface for selective recognition in aqueous solution. Anal. Bioanal. Chem. 405, 4245–4252. 10.1007/s00216-013-6812-6 23430187

[B36] Sobiech M. Bujak P. Lulinski P. Pron A. (2019). Semiconductor nanocrystal-polymer hybrid nanomaterials and their application in molecular imprinting. Nanoscale 11, 12030–12074. 10.1039/C9NR02585E 31204762

[B37] Sobiech M. Lulinski P. Halik P. Maciejewska D. (2017). The selective response of a templated polymer for the cationic drug pentamidine: implications from molecular simulations and experimental data. RSC Adv. 7, 46881–46893. 10.1039/C7RA07590A

[B38] Sobiech M. Zolek T. Lulinski P. Maciejewska D. (2014). A computational exploration of imprinted polymer affinity based on voriconazole metabolites. Analyst 139, 1779–1788. 10.1039/c3an01721d 24516859

[B39] Speltini A. Scalabrini A. Maraschi F. Sturini M. Profumo A. (2017). Newest applications of molecularly imprinted polymers for extraction of contaminants from environmental and food matrices: a review. Anal. Chim. Acta 974, 1–26. 10.1016/j.aca.2017.04.042 28535878

[B40] Vicario A. Solari M. Felici E. Aragon L. Bertolino F. Gomez M. R. (2018). Molecular imprinting on surface of silica particles for the selective extraction of benzylparaben in flow system applied to cosmetics and water samples. Microchem. J. 142, 329–334. 10.1016/j.microc.2018.06.031

[B41] Wang H. Yuan L. Zhu H. Jin R. Xing J. (2019). Comparative study of capsaicin molecularly imprinted polymers prepared by different polymerization methods. J. Polym. Sci. Pol. Chem. 57, 157–164. 10.1002/pola.29281

[B42] Wang H. B. Ma F. Zhou L. Qian Y. Sun Y. S. Xu Y. K. (2019). Polar surface dominated octagonal Sn doped ZnO nanowires and their room-temperature photoluminance properties. Appl. Surf. Sci. 476, 265–270. 10.1016/j.apsusc.2018.12.282

[B43] Wang S. Li X. Wu H. Tian Z. Xin Q. He G. (2016). Advances in high permeability polymer-based membrane materials for CO_2_ separations. Energy Environ. Sci. 9, 1863–1890. 10.1039/C6EE00811A

[B44] Wang X. Wang X. Guo Y. (2017). Rapidly simultaneous determination of six effective components in *Cistanche tubulosa* by near infrared spectroscopy. Molecules 22, 843–843. 10.3390/molecules22050843 28534831 PMC6154300

[B45] Wang Y. Wang Y. Liu H. (2019). A novel fluorescence and SPE adsorption nanomaterials of molecularly imprinted polymers based on quantum dot-grafted covalent organic frameworks for the high selectivity and sensitivity detection of ferulic acid. Nanomaterials 9:305. 10.3390/nano9020305 30813422 PMC6409819

[B46] Wang Y. J. Zhou S. M. Xu G. Gao Y. Q. (2015). Interference of phenylethanoid glycosides from *Cistanche tubulosa* with the MTT assay. Molecules 20, 8060–8071. 10.3390/molecules20058060 25951003 PMC6272201

[B47] Wu C. J. Chien M. Y. Lin N. H. Lin Y. C. Chen W. Y. Chen C. H.. (2019). Echinacoside isolated from *Cistanche tubulosa* putatively stimulates growth hormone secretion via activation of the ghrelin receptor. Molecules 24, 720–720. 10.3390/molecules24040720 30781558 PMC6413234

[B48] Wu Y. Ma Y. Pan J. Gu R. Luo J. (2017). Porous and magnetic molecularly imprinted polymers via pickering high internal phase emulsions polymerization for selective adsorption of iambda-cyhalothrin. Front. Chem. 5:18. 10.3389/fchem.2017.00018 28401145 PMC5368171

[B49] Xiao D. Jiang Y. Bi Y. (2018). Molecularly imprinted polymers for the detection of illegal drugs and additives: a review. Microchim. Acta. 185:247. 10.1007/s00604-018-2735-4 29619574

[B50] Xu H. T. Zhang C. G. He Y. Q. Shi S. S. Wang Y. L. Chou G. X. (2019). Phenylethanoid glycosides from the Schnabelia nepetifolia (Benth.) PDCantino promote the proliferation of osteoblasts. Phytochemistry 164, 111–121. 10.1016/j.phytochem.2019.05.003 31125861

[B51] Xu J. Medina-Rangel P. X. Haupt K. Tse Sum Bui B. (2017). Guide to the preparation of molecularly imprinted polymer nanoparticles for protein recognition by solid-phase synthesis. Method Enzymol. 590, 115–141. 10.1016/bs.mie.2017.02.004 28411635

[B52] Yan Y. Song Q. Chen X. Li J. Li P. Wang Y.. (2017). Simultaneous determination of components with wide polarity and content ranges in *Cistanche tubulosa* using serially coupled reverse phase-hydrophilic interaction chromatography-tandem mass spectrometry. J. Chromatogr. A 1501, 39–50. 10.1016/j.chroma.2017.04.034 28476319

[B53] Yang J. Li Y. Huang C. Jiao Y. Chen J. (2018). A phenolphthalein-dummy template molecularly imprinted polymer for highly selective extraction and clean-up of bisphenol a in complex biological, environmental and food samples. Polymers 10:1150. 10.3390/polym10101150 30961075 PMC6403629

[B54] Yang J. Xu H. Wu S. Ju B. Zhu D. Yan Y. (2017). Preparation and evaluation of microemulsion-based transdermal delivery of *Cistanche tubulosa* phenylethanoid glycosides. Mol. Med. Rep. 15, 1109–1116. 10.3892/mmr.2017.6147 28138704 PMC5367374

[B55] Yesilova E. Osman B. Kara A. Ozer E. T. (2018). Molecularly imprinted particle embedded composite cryogel for selective tetracycline adsorption. Separ. Purif. Technol. 200, 155–163. 10.1016/j.seppur.2018.02.002

[B56] Yoshikawa M. Tharpa K. Dima S. O. (2016). Molecularly imprinted membranes: past, present, and future. Chem. Rev. 116, 11500–11528. 10.1021/acs.chemrev.6b00098 27610706

[B57] Yu M. Wu L. Miao J. Wei W. Liu A. Liu S. (2019). Titanium dioxide and polypyrrole molecularly imprinted polymer nanocomposites based electrochemical sensor for highly selective detection of p-nonylphenol. Anal. Chim. Acta 1080, 84–94. 10.1016/j.aca.2019.06.053 31409478

[B58] Zhang H. Dai B. Wang X. Li W. Han Y. Gu J. (2013). Non-mercury catalytic acetylene hydrochlorination over bimetallic Au-Co(III)/SAC catalysts for vinyl chloride monomer production. Green Chem. 15, 829–836. 10.1039/c3gc36840h

[B59] Zhang H. Guo R. Hou J. Wei Z. Li X. (2016). Mixed-matrix membranes containing carbon nanotubes composite with hydrogel for efficient CO_2_ separation. ACS Appl. Mater. Interfaces 8, 29044–29051. 10.1021/acsami.6b09786 27723300

[B60] Zhang H. Guo R. Zhang J. Li X. (2018a). Facilitating CO_2_ transport across mixed matrix membranes containing multifunctional nanocapsules. ACS Appl. Mater. Interfaces 10, 43031–43039. 10.1021/acsami.8b15269 30452220

[B61] Zhang H. Tian H. Zhang J. Guo R. Li X. (2018b). Facilitated transport membranes with an amino acid salt for highly efficient CO_2_ separation. Int. J. Greenh. Gas Con. 78, 85–93. 10.1016/j.ijggc.2018.07.014

[B62] Zhang Y. Lu Y. Zhong J. Li W. Wei Q. Wang K. (2019). Molecularly imprinted polymer microspheres prepared via the two-step swelling polymerization for the separation of lincomycin. J. Appl. Polym. Sci. 136:47938 10.1002/app.47938

[B63] Zhao F. Wang S. She Y. Zhang C. Zheng L. Jin M.. (2017). Subcritical water extraction combined with molecular imprinting technology for sample preparation in the detection of triazine herbicides. J. Chromatogr. A 1515, 17–22. 10.1016/j.chroma.2017.06.011 28789799

